# Factors influencing self-regulatory fatigue in patients undergoing chemotherapy for gynecologic cancer: a cross-sectional study

**DOI:** 10.3389/fpsyt.2024.1273151

**Published:** 2024-04-25

**Authors:** Chunhui Lin, Fengzhi Zhang, Fangfang Yang, Yuanting Lin, Tian Tian, Kaige Shi, Manman Li, Xiaoxue Li

**Affiliations:** ^1^ Department of Nursing, The Third Affiliated Hospital of Zhengzhou University, Zhengzhou, China; ^2^ Department of Gynecology, The Third Affiliated Hospital of Zhengzhou University, Zhengzhou, China; ^3^ Pediatric Rehabilitation Department, The Third Affiliated Hospital of Zhengzhou University, Zhengzhou, China; ^4^ Department of Gynecology, Henan Cancer Hospital & The Affiliated Cancer Hospital of Zhengzhou University, Zhengzhou, China

**Keywords:** genital neoplasms, female, self-regulatory fatigue, social support, resilience, psychological, self-efficacy

## Abstract

**Objective:**

To understand the current status of self-regulatory fatigue among gynecologic cancer chemotherapy patients and explore influencing factors

**Methods:**

Using convenient sampling, a total of 232 gynecological cancer chemotherapy patients from two tertiary hospitals in Zhengzhou, Henan, China, were selected as study subjects from February 2023 to April 2023. General information questionnaire, Self-Regulatory Fatigue Scale (SRF-S), Strategies Used by People to Promote Health (SUPPH) Scale, Connor-Davidson resilience scale (CD-RISC) and Perceived Social Support Scale (PSSS) were employed for data collection. The data were analyzed using SPSS 26.0 software. Chi-square test and binary logistic regression were executed to explore the correlates of self-regulatory fatigue, the significance level (*α*) was set at 0.05.

**Results:**

The self-regulatory fatigue score of the 232 patients was 44 (36, 56). Binary logistic regression analyses revealed significant associations, demonstrating that residing in urban areas (*OR*=0.241, *P*=0.015), having no comorbidities (*OR*=0.158, *P*=0.015), increased perceived social support (*OR*=0.937, *P*=0.001), strong self-efficacy *(OR*=0.959, *P*=0.021), and heightened psychological resilience (*OR*=0.895, *P*<0.001) acted as protective factors against self-regulatory fatigue (*P* < 0.05).

**Conclusion:**

Patients residing in rural areas, having more than two comorbidities, lower self-efficacy and psychological resilience levels, and lower perceived social support are indicative of higher levels of self-regulatory fatigue. Identifying these influencing factors can provide references and support for developing individualized support and intervention measures to improve patients’ physical and mental well-being.

## 1 Introduction

According to data released by the International Agency for Research on Cancer in 2020, there are approximately 1.335 million new cases of gynecological malignancies worldwide each year, resulting in around 540,000 deaths. In China, the annual incidence of new cases is about 210,000, leading to 70,000 to 80,000 deaths, and there is a rising trend in the incidence rate (1, 2). Chemotherapy as one of the therapeutic approaches for gynecological malignancies, not only improves cancer patients’ survival rates but also presents various challenges to their functional status and quality of life (3, 4).

Research indicates that gynecological cancer patients undergoing chemotherapy not only experience common symptoms seen in most cancer patients, such as fatigue, sleep disturbances, peripheral neuropathy, anxiety, depression, and stigma (5, 6), but also unique symptoms like diminished femininity, sexual dysfunction, menopausal symptoms, and decreased fertility (7, 8). During chemotherapy, patients not only need to monitor and identify their adverse symptoms but also continuously adjust and cope with the negative impacts of chemotherapy on their well-being (9, 10). Faced with the combined effects of chemotherapy-induced toxicities, psychological and social stressors, the burden of symptoms, and psychological strain during chemotherapy might deplete patients’ self-regulation resources and weaken their ability to self-regulate and cope (11).

Self-depletion theory (12–14) posits that self-regulation resources are limited, and individuals expend limited self-control resources when engaging in self-regulatory behaviors. This can lead to temporary decreases in willpower and motivation, further manifesting as self-depletion phenomena where cognitive, emotional, and behavioral regulatory abilities decline. Self-regulation fatigue affects patients’ dietary compliance (15), reduces their proactive engagement in disease self-management (16) and diminishes their quality of life (17), thereby significantly threatening their physical and mental well-being.

Studies have shown that populations such as students (18),nurses (19), individuals with chronic illnesses (20) and cancer patients (17) experience self-regulation fatigue. Factors such as educational level (21), family economic status (22), place of residence, and medical insurance (23) have been proven to significantly influence the level of self-regulation fatigue. Additionally, the diversity in social support systems may impact patients’ levels of self-regulation fatigue (21, 24), while a strong sense of self-efficacy (25) and psychological resilience (22) contribute to better coping with self-regulation fatigue. In-depth exploration of self-regulation fatigue across different cultural and social backgrounds aids in comprehensively understanding its influencing factors.

However, research on self-regulation fatigue among gynecologic malignant tumor chemotherapy patients is currently insufficient, and studies investigating the specific impacts of the aforementioned factors on this population are yet to be fully explored. Therefore, this study aims to delve into the current status of self-regulation fatigue among this population and explore the effects of demographic, psychological resilience, self-efficacy, social support, and other variables on self-regulation fatigue in gynecologic malignant tumor chemotherapy patients.

## 2 Materials and methods

### 2.1 Design, setting, and participants

This study is a cross-sectional survey conducted from February 2023 to April 2023(registration number: 2023-061-01). Convenient sampling method was employed to select gynecological malignancy chemotherapy patients from two tertiary hospitals in Zhengzhou, China, as the survey participants. Paper-based questionnaires were administered, including General information questionnaire, Self-Regulatory Fatigue Scale (SRF-S), Strategies Used by People to Promote Health (SUPPH) Scale, Connor-Davidson resilience scale (CD-RISC) and Perceived Social Support Scale (PSSS). The sample size was calculated according to the principle of Kendall estimation of sample size (26). This demonstrated that the sample size was 5~10 times that of the independent variables. There were 28 independent variables in this study. Considering a 20% sample loss rate, the minimum sample size was [28 × 5 × (1 - 20%)] = 175. A total of 232 eligible samples were included for data analysis. All participants provided informed consent and agreed to take part in the study. The study’s criteria for inclusion were outlined as follows (1): Clinicopathologically confirmed tumors of the gynecologic oncology, such as cervical, endometrial, ovarian, and fallopian tube cancers (2); Age ≥ 18 years (3); Initial diagnosis (4); Undergone chemotherapy at least once (5); Clear consciousness and normal abilities in listening, speaking, reading, and writing (6); Signed informed consent and willingness to participate in the questionnaire survey. Exclusion criteria (1): Cases where family members request concealment of the illness (2); Concurrent presence of other malignant tumors (3); History of mental illness or psychological disorders.

### 2.2 Measurements

#### 2.2.1 Disease demographics of participants

Based on prior researches (20, 27, 28), a comprehensive demographic questionnaire was developed for patients. The questionnaire encompasses participant information including age, marital status, BMI, educational background, residence, average monthly household income, medical payment methods, primary caregivers, occupation, comorbidities, other concurrent illnesses, type of cancer, cancer stage, chemotherapy cycles, duration of cancer diagnosis(months) and chemotherapy regimen.

#### 2.2.2 Self-regulatory fatigue scale

The Self-Regulatory Fatigue Scale, formulated by Nes (29), this scale is designed to assess the extent of individual resource depletion. The scale is composed of three distinct dimensions and incorporates a total of sixteen items. These dimensions encompass cognitive control (six items), emotional control (five items), and behavioral control (five items). Responses to each item are gauged using a 5-point Likert scale, ranging from “Strongly Disagree” to “Strongly Agree,” with corresponding scores spanning from 1 to 5. The cumulative score, which can range from 16 to 80, serves as an indicator of the extent of self-regulatory resource depletion and fatigue experienced by patients. The Chinese version of the Self-Regulatory Fatigue Scale exhibited good content validity (0.677) and reliability (Cronbach’s alpha = 0.84) in its assessment (30). The scale utilized in the present study demonstrated a Cronbach’s alpha of 0.895.

#### 2.2.3 Perceived social support scale

The Perceived Social Support Scale, developed by Zimet (31), and the Chinese version of the Scale (32) was employed in this study to assess the level of social support perceived by patients. The scale comprises three distinct dimensions: family support (items 3, 4, 8, 11), friend support (items 6, 7, 9, 12), and other support (items 1, 2, 5, 10), totaling 12 items. Responses are recorded on a 7-point Likert scale ranging from 1 to 7, corresponding to gradations from “Strongly Disagree” to “Strongly Agree,” resulting in a possible total score range of 12 to 84. Elevated cumulative scores signify heightened perceived social support levels within the individual. The Chinese version of the Perceived Social Support Scale (PSSS) has exhibited favorable reliability and validity among patients (33, 34). The present study’s iteration of this scale yielded a Cronbach’s α coefficient of 0.934.

#### 2.2.4 Connor-Davidson resilience scale

The resilience scale was developed by Connor and Davidson (35), and for this study, we employed the Chinese version of the scale translated by Yu et al. (36). The scale employs a 5-level Likert rating system, encompassing three dimensions and consisting of 25 items: Resilience (items 11, 12, 13, 14, 15, 16, 17, 18, 19, 20, 21, 22, 23), Strength (items 1, 5, 7, 8, 9, 10, 24, 25), and Optimism (items 2, 3, 4, 6). Responses are rated on a scale from 0 to 4, representing levels of “not at all,” “rarely,” “sometimes,” “often,” and “almost always,” correspondingly. The cumulative score spans from 0 to 100, with elevated scores denoting heightened psychological resilience. Previous research has demonstrated the Chinese version of the scale’s strong reliability and validity (36, 37). For this investigation, the Cronbach’s alpha coefficient for the scale was determined as 0.962.

#### 2.2.5 Strategies used by people to promote health scale

The Strategies Used by People to Promote Health (SUPPH) Scale, developed by Lev (38), is utilized to measure patients’ self-efficacy. In this study, we employed the Chinese version of the scale translated by Qian (39). The scale comprises 28 items, categorized into three dimensions: Self-Relief Dimension (items 1-6, 8, 9, 13, 14), which assesses individuals’ ability to regulate self-pressure; Self-Decision Dimension (items 10-12), evaluating individuals’ confidence in participating in disease treatment decisions; and Positive Attitude Dimension (items 7, 15-28), which assesses individuals’ positive outlook on treatment outcomes and life. The scale utilizes a 5-point Likert rating system, ranging from “no confidence” to “very confident,” with scores assigned from 1 to 5. Scores range from 28 to 140, with higher scores indicating greater self-management efficacy. The Chinese version of the Health Promotion Strategies Scale displayed robust reliability and satisfactory validity, rendering it applicable across various research investigations (40, 41). The study exhibited a Cronbach’s alpha coefficient of 0.983.

### 2.3 Data collection

**Figure 1:**
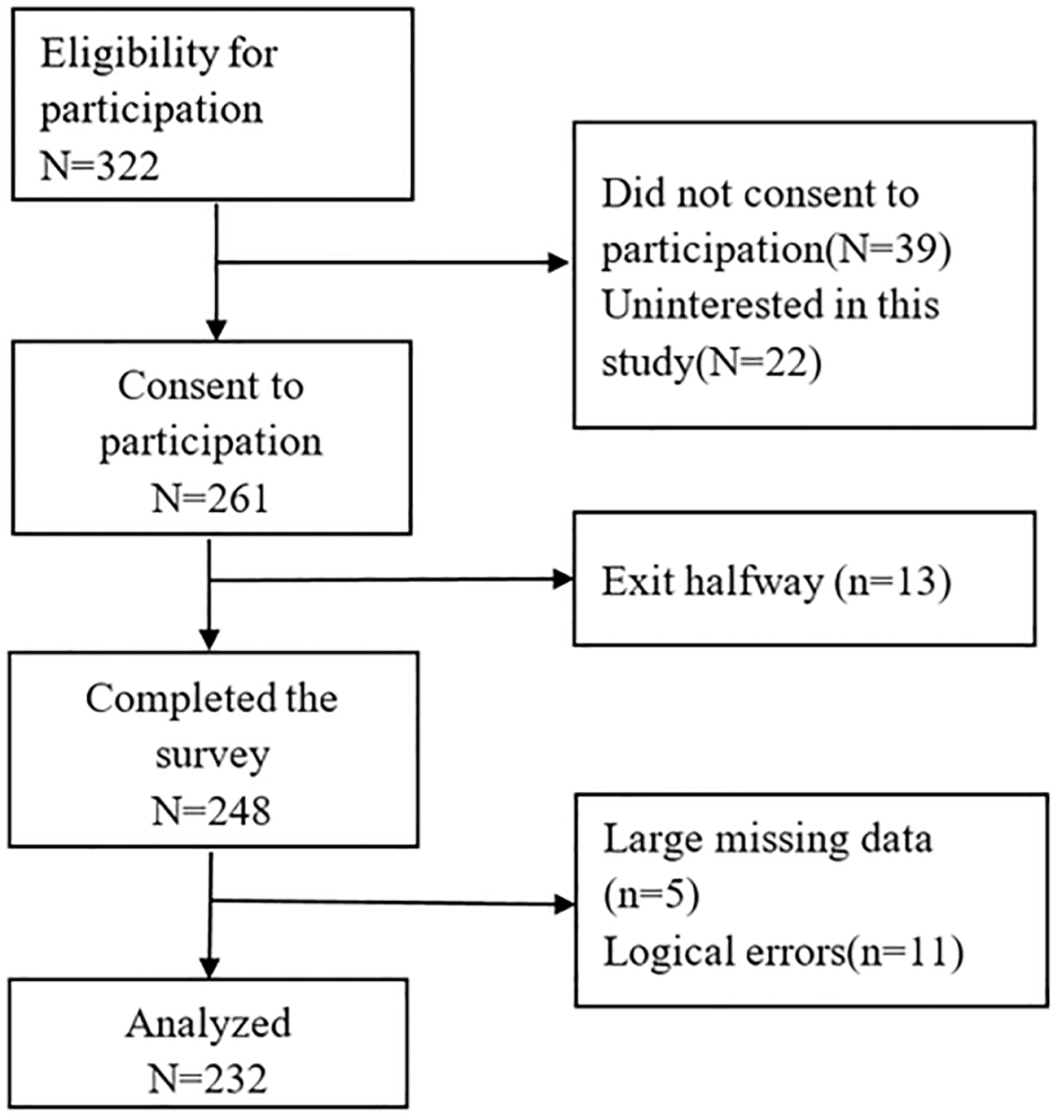
A flow chart of the participants.

Following ethical approval from the hospital’s ethics committee (No: 2023-061-01) and informed consent from participants, paper-based surveys were conducted by the researcher and two trained and qualified surveyors. Prior to the survey, participants were briefed about the study’s purpose, significance, questionnaire completion process, and instructions, and they signed informed consent forms. The survey was conducted using paper-based questionnaires and standardized instructions. All completed questionnaires were collected on-site and examined for completeness. Out of 261 distributed questionnaires, 248 were successfully retrieved, and the final analysis was based on 232 valid responses. **Figure 1** depicts the participants’ flowchart.

### 2.4 Data analysis

Statistical analysis employed SPSS version 26.0, with statistical significance established at *P* < 0.05. Descriptive statistics, including frequencies and percentages (%), were used for categorical data. For normally distributed quantitative data, descriptive statistics such as mean and standard deviation are used. For variables such as self-regulation fatigue and social support, which displayed non-normal distribution (validated via the Kolmogorov-Smirnov test), median (Me) and quartiles (*Q*25, *Q*75) were used to describe continuous data. Between-group comparisons were assessed using the chi-square test. Self-regulation fatigue was taken as the dependent variable, categorized by its median score into low and high self-regulation fatigue groups. Binary logistic regression was employed to analyze factors influencing self-regulation fatigue levels, with results presented as odds ratios (*OR*) and 95% confidence intervals.

## 3 Results

### 3.1 Participant characteristics

A total of 232 gynecological malignancy chemotherapy patients were included in this study. Among them, 90.09% were aged 35 and above, 89.66% were married, 56.03% had abnormal weight, 71.12% with education level of junior high school and above, 59.91% resided in urban areas, 60.34% had a monthly household income below 3000, 64.22% were covered by the New Rural Cooperative Medical System, and 57.33% had their spouse as the primary caregiver. The comprehensive demographic and disease characteristics of the participants are outlined in **Table 1**.

**Table 1:** Distribution of Demographic and Illness Characteristics.

Variable	Categories	Frequency (n)	Percent (%)
Age (years)	18-35	23	9.91
	36-45	46	19.83
	46-55	76	32.76
	>55	87	37.5
Marital status	Unmarried	12	5.17
	Married	208	89.66
	Divorce or other	12	5.17
BMI (kg/m^2^)	Underweight	18	7.76
	Normal weight	102	43.97
	Overweight	91	39.22
	Obesity	21	9.05
Educational background	Primary and lower	67	28.88
	junior	85	36.64
	High school or junior college	43	18.53
	College and above	37	15.95
Residence	Urban	139	59.91
	Rural	113	48.71
Monthly household income (yuan)	1000	60	25.86
	1001-3000	80	34.48
	3001-5000	56	24.14
	>5000	36	15.52
Medical Payment Methods	New Rural Cooperative Medical Insurance	149	64.22
	Resident health insurance	26	11.21
	Employee health insurance	57	24.57
Caregivers	parents	14	6.03
	Spouse	133	57.33
	Child	66	28.45
	other	19	8.19
Occupation	Farmer	47	20.26
	Worker	94	40.52
	Staff	28	12.07
	others	47	20.26
	Resignation/retirement	16	6.90
Comorbidities	None	118	50.86
	One	63	27.16
	Two or more	51	21.98
Number of combined other diseases	None	172	74.14
	One	45	19.40
	Two or more	15	6.47
Type of Cancer	Cervical	79	34.05
	Endometrium	30	12.93
	Ovary	108	46.55
	Other	15	6.47
Cancer Stage	I	58	25.00
	II	41	17.67
	III	92	39.66
	IV	41	17.67
No of chemotherapy cycles	1	42	18.10
	2	68	29.31
	3	50	21.55
	4	21	9.05
	5	14	6.03
	6	9	3.88
	>6	28	12.07
Duration of cancer diagnosis(months)	<3	60	25.86
	4~6	76	32.76
	7~12	42	18.10
	>12	54	23.28
Chemotherapy regimen	TC	159	68.5
	TP	13	5.6
	DP	30	12.9
	GP	9	3.9
	Other	21	9.1

### 3.2 Univariate analysis

Differences in participants’ self-regulation fatigue based on demographic and disease characteristics are shown in **Table 2**. Chi-square tests revealed statistically significant differences in self-regulation fatigue levels for variables such as education level (*χ^2^* = 42.079, *P*<0.001), residence (*χ* ^2^ = 11.801, *P*=0.001), occupation (*χ* ^2^ = 11.935, *P*=0.018), comorbidities (*χ* ^2^ = 27.376, *P*<0.001), combined other diseases (*χ* ^2^ = 10.417, *P*=0.005), and type of Cancer (*χ* ^2^ = 7.900, *P*=0.048).

**Table 2:** Univariate analysis of the participants (n = 232).

Variable	Categories	Low(N=111)	High(N=121)	*χ²*	*P* value
Age (years)	18-35	13 (56.5%)	10 (43.5%)	3.915	0.271
	36-45	23 (50.0%)	23 (50.0%)		
	46-55	40 (52.6%)	36 (47.4%)		
	>55	35 (40.2%)	52 (59.8%)		
Marital status	Unmarried	7 (58.3%)	5 (41.7%)	2.471	0.291
	Married	96 (46.2%)	112 (53.8%)		
	Divorce or other	8 (66.7%)	4 (33.3%)		
BMI (kg/m^2^)	Underweight	11 (61.1%)	7 (38.9%)	3.525	0.317
	Normal weight	46(45.1%)	56(54.9%)		
	Overweight	41(45.1%)	50(54.9%)		
	Obesity	13(61.9%)	8(38.1%)		
Educational background	Primary and lower	12(17.9%)	55(82.1%)	42.079	<0.001
	junior	42(49.4%)	43(50.6%)		
	High school or junior college	32(74.4%)	11(25.6%)		
	College and above	25(67.6%)	12(32.4%)		
residence	Urban	70(58.8%)	49(41.2%)	11.801	0.001
	Rural	41(36.3%)	72(63.7%)		
Monthly household income (yuan)	1000	23(38.3%)	37(61.7%)	5.710	0.127
	1001-3000	36 (45.0%)	44 (55.0%)		
	3001-5000	30 (53.6%)	26 (46.4%)		
	>5000	22 (61.1%)	14 (38.9%)		
Medical Payment Methods	New Rural Cooperative Medical Insurance	63 (42.3%)	86 (57.7%)	5.406	0.067
	Resident health insurance	14 (53.8%)	12(46.2%)		
	Employee health insurance	34(59.6%)	23(40.4%)		
Caregivers	Parents	8(57.1%)	6(42.9%)	3.173	0.366
	Spouse	57(42.9%)	76(57.1%)		
	Child	36(54.5%)	30(45.5%)		
	Other	10(52.6%)	9(47.4%)		
Occupation	Farmer	23(48.9%)	24 (51.1%)	11.935	0.018
	Worker	34 (36.2%)	60 (63.8%)		
	Staff	17 (60.7%)	11 (39.3%)		
	others	30 (63.8%)	17 (36.2%)		
	Resignation/retirement	7 (43.8%)	9 (56.3%)		
Comorbidities	None	74 (62.7%)	44 (37.3%)	27.376	<0.001
	One	27 (42.9%)	36 (57.1%)		
	Two or more	10 (19.6%)	41 (80.4%)		
Number of combined other diseases	None	93 (54.1%)	79 (45.9%)	10.417	0.005
	One	13(28.9%)	32(71.1%)		
	Two or more	5(33.3%)	10(66.7%)		
Type of Cancer	Cervical	40(50.6%)	39(49.4%)	7.900	0.048
	Endometrium	13(43.3%)	17(56.7%)		
	Ovary	46(42.6%)	62(57.4%)		
	other	12(80.0%)	3(20.0%)		
Cancer Stage	I	26(44.8%)	32(55.2%)	6.996	0.072
	II	27(65.9%)	14(34.1%)		
	III	42(45.7%)	50(54.3%)		
	IV	16(39.0%)	25(61.0%)		
No of chemotherapy cycles	1	20(47.6%)	22(52.4%)	0.925	0.988
	2	31(45.6%)	37(54.4%)		
	3	25(50.0%)	25(50.0%)		
	4	11(52.4%)	10(47.6%)		
	5	7(50.0%)	7(50.0%)		
	6	5(55.6%)	4(44.4%)		
	>6	12(42.9%)	16(57.1%)		
Duration of cancer diagnosis(months)	<3	23(38.3%)	37(61.7%)	3.981	0.263
	4~6	42(55.3%)	34(44.7%)		
	7~12	21(50.0%)	21(50.0%)		
	>12	25(46.3%)	29(53.7%)		
Chemotherapy regimen	TC	80 (50.3%)	79 (49.7%)	2.205	0.712
	TP	4 (30.8%)	9 (69.2%)		
	DP	13 (43.3%)	17 (56.7%)		
	GP	4 (44.4%)	5 (55.6%)		
	Other	10 (47.8%)	11 (52.2%)		

### 3.3 Correlation analysis

In this study, the self-regulation fatigue score was 44 (36, 42), perceived social support score was 53 (42, 63.75), self-efficacy score was 82.43± 24.69, and resilience score was 56.57 ± 16.88. Spearman correlation analysis revealed significant negative correlations between self-regulation fatigue and perceived social support (*r*=-0.534, *P*<0.01), self-efficacy (*r*=-0.752, *P*<0.01), and psychological resilience (*r*=-0.775, *P*<0.01). Further information can be found in **Table 3**.

**Table 3:** Spearman correlation analysis.

Variable	Score, *M* (*P*25, *P*75)	Perceived Social Support	Self-efficacy	Psychological resilience	Self-regulatory fatigue
Perceived Social Support	53 (42,63.75)				
Self-efficacy	82.43±24.69	0.383^**^			
Psychological resilience	56.57±16.88	0.379^**^	0.851^**^		
Self-regulatory fatigue	44 (36,56)	-0.534^**^	-0.752^**^	-0.775^**^	1

^**^P<0.01,2-tailed.

### 3.4 Factors affecting self-regulation fatigue

Using self-regulation fatigue as the dependent variable and classifying variables with statistically significant results from univariate analysis and Spearman correlation analysis based on whether their median score indicated high or low levels of self-regulation fatigue, binary logistic regression analysis revealed that residing in urban areas (*OR*=0.241, *P*=0.015), absence of comorbidities (*OR*=0.158, *P*=0.015), higher perceived social support (*OR*=0.937, *P*=0.001), stronger self-efficacy (*OR*=0.959, *P*=0.021), and greater resilience (*OR*=0.895, *P*<0.001) were associated with lower levels of self-regulation fatigue (*P* < 0.05). Refer to **Table 4** for details.

**Table 4:** A binary logistic regression analysis of factors associated with self-regulatory fatigue (n = 232).

Variables	*B*	*OR*	*OR* (95% *CI*)	*P*-value
Educational background				0.674
Primary and lower	-0.273	0.761	0.115-5.038	0.777
junior	-0.456	0.634	0.128-3.127	0.575
High school or junior college	-1.018	0.361	0.066-1.965	0.239
College and above	Ref			
Residence				
Urban	-1.423	0.241	0.077-0.757	0.015^*^
Rural	Ref			
Occupation				0.267
Farmer	0.918	2.504	0.293-21.348	0.402
Worker	-0.653	0.521	0.064-4.222	0.541
Staff	1.107	3.027	0.302-30.352	0.346
Others	0.521	1.684	0.203-13.998	0.63
Resignation/retirement	Ref			
Comorbidities				0.049
None	-1.847	0.158	0.036-0.696	0.015^*^
One	-1.305	0.271	0.056-1.307	0.104
Two or more	Ref			
Combined other diseases				
None	0.472	1.604	0.219-11.702	0.643
One	0.862	2.368	0.261-21.553	0.444
Two or more	Ref			
Type of Cancer				0.388
Cervical	1.249	3.491	0.538-22.641	0.191
Endometrium	0.766	2.151	0.255-18.166	0.482
Ovary	1.488	4.427	0.689-28.446	0.117
Other	Ref			
**Perceived Social Support**	-0.065	0.937	0.903-0.973	0.001^**^
**Self-efficacy**	-0.042	0.959	0.926-0.994	0.021^*^
**Psychological resilience**	-0.111	0.895	0.845-0.948	< 0.001^**^
Hosmer-Lemeshow test	*P*= 0.674			

^*^P<0.05, ^**^P<0.01.

## 4 Discussion

In this study, patients’ self-regulation fatigue scores were 44 (36, 42), which were higher than China’s norm (36.5 ± 8.9) (30), yet lower than findings in studies of cervical cancer radiotherapy patients (54.59 ± 15.09) (43) and breast cancer chemoradiotherapy patients(51.77 ± 13.48) (22). The disparities might be attributed to differences in cancer type and severity. Our study included gynecological malignancy chemotherapy patients, who potentially encounter distinct symptoms across various diseases. The self-regulation resource model (44) suggests that individuals have limited self-regulatory resources over time, which can be depleted faster by experiencing symptom distress, negative events, and stress (45), ultimately leading to self-regulation fatigue. Patients undergoing treatment for gynecological malignancies often face protracted treatment regimens, necessitating extended recovery periods. Patients need to manage symptoms themselves during chemotherapy, and the side effects and symptom distress may excessively deplete their self-regulatory resources, impairing their self-regulation function and resulting in self-regulation fatigue. Additionally, gynecological malignancy patients may experience unique symptoms such as reproductive system damage, perimenopausal symptoms, diminished female characteristics, and reduced fertility during treatment (46). In such cases, emotions like anxiety, depression, and psychological distress are more common (47), potentially diminishing patients’ cognitive control abilities (48), placing them in a state of self-regulation fatigue. Self-regulation fatigue impairs patients’ self-management abilities, weakens health-promoting behaviors, and diminishes quality of life (17, 20, 49). Thus, healthcare professionals should regularly assess and monitor the levels of self-regulation fatigue in gynecological malignancy chemotherapy patients. Timely detection and management of self-regulation fatigue contribute to improving their quality of life and enhancing health outcomes.

Our study revealed that patients residing in urban areas exhibited lower levels of self-regulation fatigue compared to those in rural areas, consistent with the findings of Ji’s research on coronary heart disease patients (27). The variance might stem from disparities in healthcare resources between urban and rural regions. Urban patients have access to better medical resources and support systems, facilitating active disease coping and minimizing psychological resource depletion (22). On the other hand, patients in rural areas may rely more on self-regulation strategies. This underscores the need for healthcare professionals to pay attention to self-regulation fatigue among rural patients, providing them with specialized guidance and support to enhance their ability in managing self-regulation fatigue.

Our study discovered that patients without complications experienced lower levels of self-regulation fatigue compared to those with two or more complications, aligning with the findings of Zhang (28). This could be attributed to ongoing symptom distress limiting patients’ activity and rendering symptom management ineffective, resulting in greater depletion of psychological resources (45), This highlights the necessity for healthcare providers to tailor personalized interventions based on patient symptoms, aiming to reduce self-regulation fatigue.

Our research also observed that higher levels of perceived social support among gynecological malignancy chemotherapy patients were associated with lower levels of self-regulation fatigue, consistent with the results of Zhang’s study on 942 nurses (21). Perceived social support refers to an individual’s emotional experience and satisfaction with being respected, supported, and understood in society, closely tied to their subjective feelings (50). In the face of multiple stressors such as physiological and psychological challenges, strong social support can offer positive coping strategies, aiding patients in better handling stressful events (51). Additionally, elevated levels of social support could serve as supplementary resources for self-regulation, reducing perceived stress levels and thereby facilitating effective self-regulation (24). Partners, being vital sources of social support for patients, contribute to enhancing patients’ psychological resilience during the treatment and recovery process, as well as improving overall quality of life (52, 53). Therefore, healthcare professionals should focus on gynecological cancer chemotherapy patients’ perception of social support. Healthcare professionals can effectively strengthen patients’ ability to cope with the challenges of chemotherapy, thereby reducing self-regulation fatigue, by providing patients with relevant professional support and encouraging them to actively seek support from family members, especially from their partners. The findings of this study indicate a significant negative correlation between patients’ self-efficacy and self-regulation fatigue. In other words, patients with higher self-efficacy exhibit greater confidence in disease recovery and experience lower levels of self-regulation fatigue. This aligns with the results of Zhang’s study on 275 rheumatoid arthritis patients (23) and Li Lu’s study on 1122 university students (54). Enhanced self-efficacy can elevate cancer patients’ positive coping levels and mitigate symptom severity (42, 55). Low self-efficacy among gynecological malignancy chemotherapy patients might lead to feelings of helplessness when dealing with treatment side effects, potentially contributing to increased self-regulation fatigue. This suggests that healthcare professionals could bolster treatment confidence and enhance self-efficacy through psychological education programs (47), thereby ameliorating self-regulation fatigue during chemotherapy.

Psychological resilience refers to a patient’s capacity to dynamically adjust their levels of psychological distress when facing adversity related to cancer, achieved through interactions with the environment (56), It holds significant importance in elevating patients’ self-care abilities and alleviating psychological distress (57, 58). The present study’s results reveal psychological resilience as a protective factor against self-regulation fatigue among gynecological malignancy chemotherapy patients. Higher levels of psychological resilience correspond to lower levels of self-regulation fatigue, consistent with Zhou’s findings on young breast cancer patients (22). As a positive psychological resource, psychological resilience might aid patients in adapting to negative emotions, promoting accurate disease perception, and rectifying maladaptive behaviors, ultimately reducing the depletion of self-regulation resources and preventing self-regulation fatigue (22, 59). Additionally, patients with higher psychological resilience tend to exhibit superior coping abilities, potentially experiencing fewer adverse reactions and symptom distress during chemotherapy (53, 60), thereby reducing the strain on psychological resources. Thus, healthcare professionals should consider enhancing individual psychological resilience, employing interventions such as positive psychology techniques (61), to improve patients’ self-regulation fatigue status.

### 4.1 Practical implications

This study investigated self-regulation fatigue and its impact on gynecological malignancy chemotherapy patients. Our findings offer valuable insights for tailoring interventions to alleviate self-regulation fatigue and improve patients’ quality of life. Furthermore, these results provide guidance for future nursing practices and research endeavors in this area.

### 4.2 Limitations of the study

This study relied on self-reported data from patients, which could introduce subjectivity to the findings. Additionally, the sample size was limited to patients from two tertiary hospitals in Henan Province, China. Future research should consider employing larger sample sizes and adopting a multi-center research design to enhance the reliability and generalizability of the results. Longitudinal and intervention studies are recommended to explore causal relationships and trends, and to validate the effectiveness of intervention measures targeting relevant factors in mitigating self-regulation fatigue among gynecological malignancy chemotherapy patients, thus ultimately enhancing their quality of life.

## 5 Conclusion

The current study underscores the need for improvement in self-regulation fatigue among gynecological malignancy chemotherapy patients. Special attention should be given to patients residing in rural areas, those with multiple complications, low self-efficacy, lower levels of psychological resilience, and limited perceived social support. Recognizing the unique self-regulation fatigue challenges faced by these patients, healthcare teams can develop personalized care plans. These plans might involve regular assessments of self-regulation fatigue levels, provision of relevant information, skills training, emotional support, and psychological counseling. Enhancing patients’ self-efficacy can effectively aid them in coping with self-regulation fatigue.

## Data availability statement

The raw data supporting the conclusions of this article will be made available by the authors, without undue reservation.

## Ethics statement

The studies involving humans were approved by the Chinese Department of Health and the Ethics Committee of Zhengzhou University’s third affiliated hospital (Approval No: 2023-061-01). The studies were conducted in accordance with the local legislation and institutional requirements. The participants provided their written informed consent to participate in this study.

## Author contributions

CL: Writing – original draft. FZ: Funding acquisition, Writing – review & editing. FY: Writing – review & editing, Investigation. YL: Writing – review & editing, Supervision, Data curation. TT: Writing – review & editing, Investigation. KS: Writing – review & editing, Validation, Supervision. ML: Writing – review & editing, Data curation. XL: Writing – review & editing, Validation, Supervision.
